# Correction: Liu et al. *LeGRXS14* Reduces Salt Stress Tolerance in *Arabidopsis thaliana*. *Plants* 2023, *12*, 2320

**DOI:** 10.3390/plants12233987

**Published:** 2023-11-27

**Authors:** Lulu Liu, Xiaofei Li, Mengke Su, Jiaping Shi, Qing Zhang, Xunyan Liu

**Affiliations:** 1 College of Life Sciences and Oceanography, Shenzhen University, Shenzhen 518060, China; liululu124@hotmail.com; 2 College of Physics and Optoelectronic Engineering, Shenzhen University, Shenzhen 518060, China; 3 College of Life and Environmental Sciences, Hangzhou Normal University, Hangzhou 310030, China; lxf17633576763@163.com (X.L.); smk155321@163.com (M.S.); sjp12302000@163.com (J.S.); zq15025721179@126.com (Q.Z.)

The authors wish to correct the following error in the original paper [1].

An additional affiliation (College of Physics and Optoelectronic Engineering, Shenzhen University, Shenzhen 518060, China) has been added to the first author Lulu Liu. Due to this change, the numbers of the rest of the affiliations of each author were updated.

The authors apologize for any inconvenience caused and state that the scientific conclusions are unaffected. This correction was approved by the Academic Editor. The original publication has also been updated.
