# Supplementary material for: LeGRXS14 Reduces Salt Stress Tolerance in Arabidopsis thaliana
Source: Plants (Basel). 2023 Jun 15;12(12):2320. doi: 10.3390/plants12122320 (PMC10305512; doi:10.3390/plants12122320)
Supplement: Supplementary file 1 [file plants-12-02320-s001.zip › plants-2359396-supplementary.pdf]

Table S1. Gene ID used in this paper.

Name	Gene ID
LeGRXS14	Solyc02g082200
AtGRXS14	AT3G54900
BnGRXS14	CDX78278
CaGRXS14	PHT90856
ClGRXS14	Cla97C11G217130
CsGRXS14	KGN61246
GmGRXS14	KRH00894
OsGRXS14	Os03t0851200
ZmGRXS14	Zm00001eb064590_P001
LeGRXS15	Solyc06g067960
AtGRXS15	AT3G15660
BnGRXS15	CDX97692
CaGRXS15	PHT79445
ClGRXS15	Cla97C10G193120
CsGRXS15	KGN52871
GmGRXS15	KRH44417
OsGRXS15	Os01t0530400
ZmGRXS15	Zm00001eb336840_P002
LeGRXS16	Solyc09g005620
AtGRXS16	AT2G38270
BnGRXS16	CDY14473
CaGRXS16	PHT72767
ClGRXS16	Cla97C10G205580
CsGRXS16	KGN58741
GmGRXS16	KRH65709
OsGRXS16	Os12t0175500
ZmGRXS16	Zm00001eb163210_P001
LeGRXS17	Solyc02g078360
AtGRXS17	AT4G04950
BnGRXS17	CDX90882
CaGRXS17	PHT90520
ClGRXS17	Cla97C01G022820
CsGRXS17	KGN49222
GmGRXS17	KRH20463
OsGRXS17	Os10t0500700
ZmGRXS17	Zm00001eb044380

Table S2. Primers used in this paper.

Name	Sequence, 5′ to 3′
LeActin-Forword	TCGTAAGGAGTGCCCTAATGCTGA
LeActin-Reverse	CAATCGCCTCCAGCCTTGTTGTAA
AtActin-Forword	AACTCTATGCAGCATTTGATCCACT
AtActin-Reverse	TGATTGCATATCTTTATCGCCATC
LeGRXS14-Forword	GCCAACCTTTCCACAACTCTAC
LeGRXS14-Reverse	CTCTCCGCTCTTGTATGCTTC
AtZAT12-Forword	AACACAAACCACAAGAGGATCA
AtZAT12-Reverse	AAGCATCAAACAATTCGCCG
AtSOS1-Forword	CTTCTTCCTCTGTGTTGTTGC
AtSOS1-Reverse	GAAGACGAATCGGTCGCTT
AtSOS2-Forword	AAGCTATGTTCGAAACTGGAAAAC
AtSOS2-Reverse	TGGATTTAAGTTGGGATCAAAACG
AtSOS3-Forword	AGAAGGGTGTGTTTGTATGGG
AtSOS3-Reverse	GAAGCTCGGGATCCTCATATC
AtTPC1-Forword	GCTCTATTGGCGTACAGGTCTTTG
AtTPC1-Reverse	GAAGAGTGTGACCATTCCATTGG
AtRbOHD-Forword	CCTATGAGCCGATGGAAAAA
AtRbOHD-Reverse	ACAATGCCAGTCCATCCTTC
AtHKT1-Forword	TTCACTTCTGTCTCTGCCATC
AtHKT1-Reverse	AGGAAGATAAGTTGGGTGTTGG
AtNHX1-Forword	TTGTATTCGGAGAGGGTGTTG
AtNHX1-Reverse	GCTTCGTGGTTTAGGTGAGTG
AtNHX2-Forword	CGCCTTGAGTGGTATCCTAAC
AtNHX2-Reverse	GAGAAACGACAAAGTAGCAAAGG
AtNHX5-Forword	CTTTTGCTGGCTCAATGTCTG
AtNHX5-Reverse	GTTCTGAAGATTCTCGGTGTCC
AtNHX6-Forword	TTAGGTGATGGATTTGAGGTGG
AtNHX6-Reverse	CCGCACTCTTATGGAACTCTC

* All primers were designed at Integrated DNA Technologies (idtdna.com)

Table S3. Lengths and preserved sequences of motifs in GRXS14.

No.	Motif Logo	Sequence	Length
Motif 1	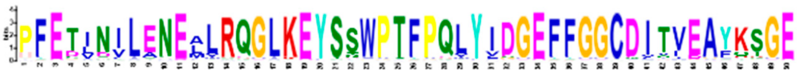	PFETINILENEALRQGLK EYSSWPTFPQLYIDGEFF GGCDITVEAYKSGE	50
Motif 2	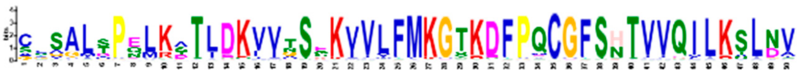	CLSALSPZLKATLDKVVT SZKVVLFMKGTKDFPQC GFSHTVVQILKSLNV	50
Motif 3	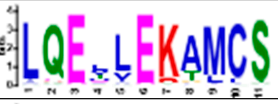	LQELLEKAMCS	11
Motif 4	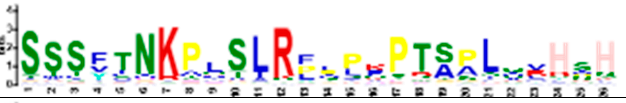	SSSFTNKPLSLRFLPKPTS PLQVHSH	26
Motif 5	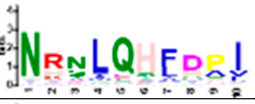	NRNLQHFDPI	10
Motif 6	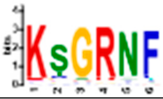	KSGRNF	6

* All motifs were founded at MEME - MEME Suite (meme-suite.org)

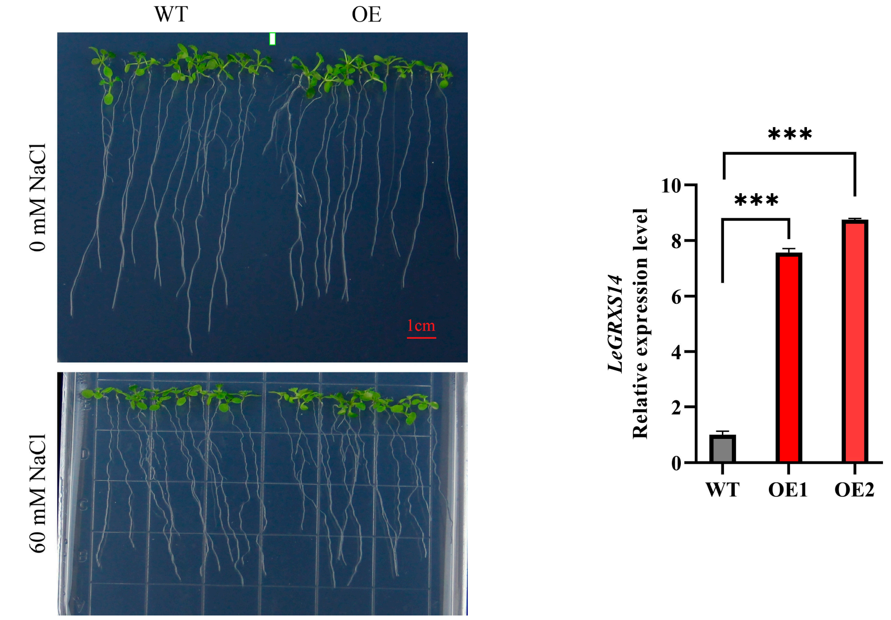

(**a**) (**b**)

Figure S1. Phenotypic analyses of OE and WT in response to Salt stress. (**a**) WT and OE were grown for 12 days on the ½ MS with or without 60 mM NaCl; (**b**) Statistic analysis of *LeGRXS14* expression in WT and OE, WT was showed with gray, OE was showed with red, the data in the figure are mean ± SD, (n = 3); *** $p <$ 0.001.

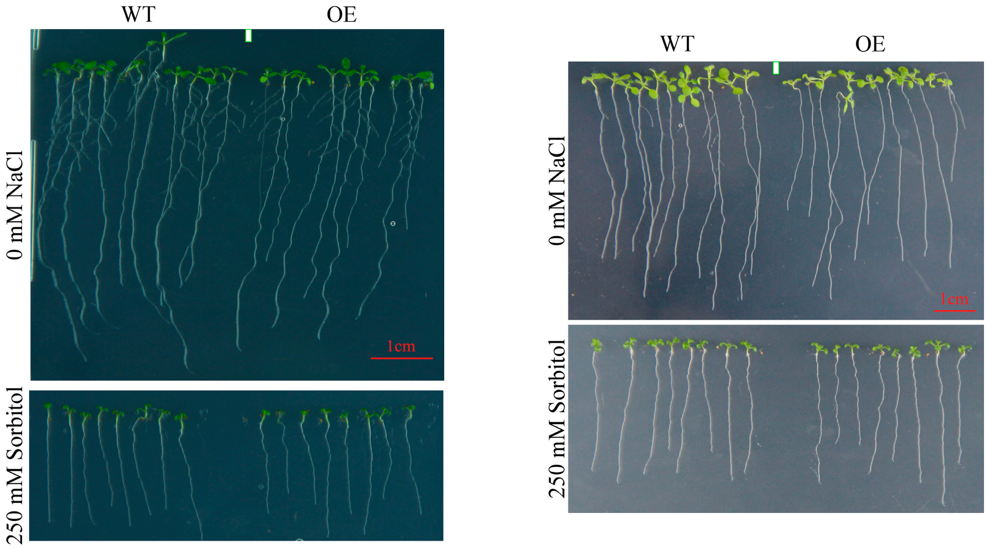

(**a**) (**b**)

Figure S2. Phenotypic analyses of OE and WT in response to osmotic stress. (**a**) WT and OE were grown for 12 days on the ½ MS with or without 250 mM sorbitol (0.2 mM Ca^{2+}); (**b**) WT and OE were grown for 8 days on the ½ MS with or without 250 mM sorbitol.

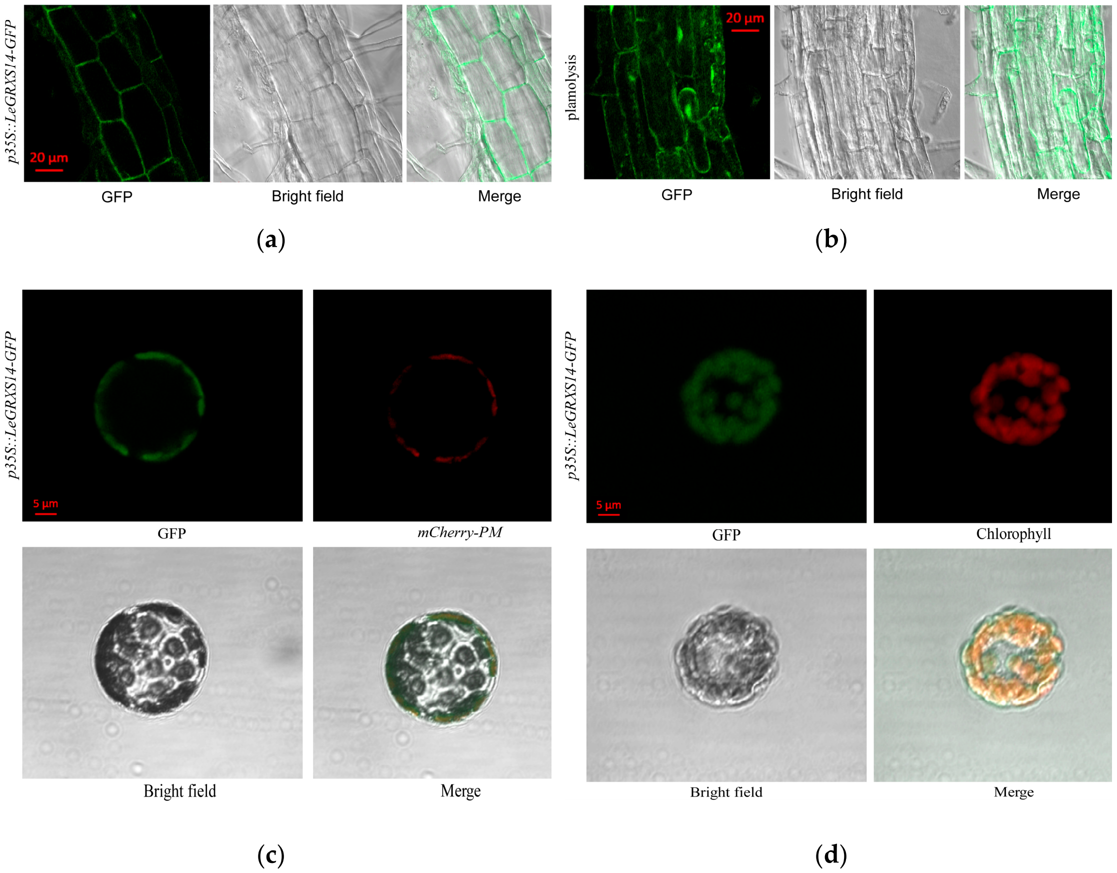

Figure S3. Subcellular localization of LeGRXS14 proteins: (**a**) Confocal micrograph of turgid periphery cells from the roots of the *p35S::LeGRXS14-GFP* construct. (**b**) Confocal micrograph of plasmolyzed cells from the roots of the *p35S::LeGRXS14-GFP* construct. Bars = 20 µm. (**c**) Subcellular localization of LeGRXS14-GFP and mCherry-plasma membrane in tobacco protoplasts. (**d**) Subcellular localization of LeGRXS14-GFP and Chlorophyll autofluorescence in transgenic *A. thaliana* leaf protoplasts containing *p35S::LeGRXS14-GFP*. Green shows GFP signals, red shows mCherry or Chlorophyll autofluorescence signals. Bars = 5 µm.
